# Seroprevalence and Epidemiological Characteristics of Severe Fever with Thrombocytopenia Syndrome in Patients with Chronic Diseases in Korea

**DOI:** 10.3390/v18020217

**Published:** 2026-02-06

**Authors:** Jongyoun Yi, Ahreum Kim, Maeng Seok Noh, Changhoon Kim, Hyun Jin Son, Mee Kyung Ko, Kye-Hyung Kim

**Affiliations:** 1 Department of Laboratory Medicine, Pusan National University School of Medicine, Busan 49241, Republic of Korea; socioliberal@pusan.ac.kr; 2 Biomedical Research Institute, Pusan National University Hospital, Busan 49241, Republic of Korea; ar_kim_@naver.com (A.K.); kchprev@pusan.ac.kr (C.K.); qeqazwsx@hanmail.net (M.K.K.); 3 Major of Bigdata Convergence, Pukyong National University, Busan 48513, Republic of Korea; msnoh@pknu.ac.kr; 4 Department of Preventive Medicine and Occupational and Environmental Medicine, Pusan National University School of Medicine, Busan 49241, Republic of Korea; 5 Department of Preventive Medicine, Dong-A University School of Medicine, Busan 49201, Republic of Korea; hjson@dau.ac.kr; 6 Department of Internal Medicine, Pusan National University School of Medicine, Busan 49241, Republic of Korea

**Keywords:** severe fever with thrombocytopenia syndrome, seroprevalence, biobank, chronic disease, epidemiology

## Abstract

Severe fever with thrombocytopenia syndrome (SFTS) is a tick-borne disease with a high mortality rate. While research has focused on high-risk rural populations and healthy individuals in endemic regions, such as Jeju Island, data on patients with underlying chronic diseases remain limited. This study aimed to evaluate the seroprevalence of SFTS virus (SFTSV) in patients with various chronic diseases across the Republic of Korea. Serum samples (n = 2948) collected from 10 regional biobanks between 2009 and 2019 were analyzed using a double-antigen sandwich enzyme-linked immunosorbent assay. The overall seroprevalence was 1.22% (36/2948). Seropositivity was significantly higher in males (1.73%) than in females (0.73%, *p* = 0.013) and increased with age (*p* = 0.001), peaking at 2.73% in individuals aged 70–79. Geographically, the highest rates were in Gyeongbuk (3.03%), Jeonnam (2.40%), and Gangwon (1.83%). Multivariable logistic regression showed older age (adjusted odds ratio 1.47 per 10-year increase, 95% confidence interval: 1.12–1.97) as the strongest independent predictor of seropositivity. Patients with hepatobiliary/pancreatic cancer (3.16%) and prostate cancer (2.50%) exhibited higher seroprevalence than those in other disease groups. SFTSV exposure is non-negligible among those with chronic diseases, particularly older males in rural provinces. Public health strategies should specifically address these vulnerable populations.

## 1. Introduction

Severe fever with thrombocytopenia syndrome (SFTS) is a tick-borne viral disease caused by *Bandavirus dabieense* (genus *Bandavirus*, family *Phenuiviridae*, order *Hareavirales*) [1]. This virus is also commonly referred to as SFTS virus (SFTSV). Since it was first identified in China in 2009 [2], SFTS has become recognized as a significant public health concern in East Asia, including South Korea [3,4,5,6] and Japan [7,8]. This is primarily because of its high case fatality rate ranging from 7.4 to 19.4%, depending on the region and patient age group [9].

In South Korea, SFTS incidence has increased since the first case was reported in 2013 [3,4,6]. Previous epidemiological studies have primarily focused on high-risk groups, such as farmers and forestry workers, or residents of highly endemic areas such as Jeju Island [10,11]. For instance, studies on Jeju Island have reported seroprevalence rates between 1.7 and 2.4% in general and agricultural populations [11,12]. However, comprehensive data regarding SFTSV seroprevalence in patients with chronic diseases (e.g., cancer, diabetes, and cardiovascular diseases) are lacking. These individuals may exhibit distinct immunological responses or exposure risks compared with the general population. Recent nationwide studies have highlighted increased mortality and morbidity risks in patients with SFTS comorbidities [13], underscoring the need for population-specific data.

Patients with chronic illnesses often visit hospitals and contribute samples to biobanks, representing a demographic that may differ significantly from healthy individuals undergoing routine health screening. Assessing seroprevalence in this group is crucial for determining the SFTSV infection burden in vulnerable populations at higher risk for severe clinical outcomes.

Therefore, this study aimed to investigate SFTSV antibody seroprevalence in a large cohort of serum samples from 10 regional biobanks in Korea. We targeted patients with chronic diseases to characterize their exposure history and epidemiological features. Samples from Jeju Island were excluded from the statistical analysis to avoid duplication, as its epidemiological characteristics were comprehensively analyzed in a separate study [12].

## 2. Materials and Methods

### 2.1. Study Population and Sample Collection

Serum samples were obtained from 10 regional Unit Biobanks of the Korea Biobank Network (KBN), providing access to a diverse patient population with underlying chronic diseases. Participating biobanks included Kangwon National University Hospital, Kyungpook National University Hospital, Gyeongsang National University Hospital, Keimyung University Dongsan Hospital, Korea University Guro Hospital, Ajou University Hospital, Jeonbuk National University Hospital, Chungnam National University Hospital, Chungbuk National University Hospital, and Chonnam National University Hwasun Hospital.

Samples collected between February 2009 and February 2019 were processed per KBN standard specimen handling guidelines, separated, aliquoted, and stored at −80 °C until distribution. The samples remained at −80 °C until immediately before enzyme-linked immunosorbent assay (ELISA) testing to ensure sample integrity. Inclusion criteria comprised patients with various chronic non-communicable diseases, specifically cancers, diabetes mellitus, and cardiovascular diseases, etc., who visited participating hospitals. Additionally, a subset of individuals who underwent general health screening (n = 100) was included in the total cohort to represent a hospital-based control group without chronic conditions. These screening samples were analyzed as part of the total population (n = 2948) but were categorized separately as ‘Others’ in subgroup analyses. Only samples with documented written consent for both human biospecimen donation and the use of human biospecimens in research were included in this study. Exclusion criteria were applied to ensure data integrity and avoid duplication. Samples were excluded if the sample ID was missing or unidentifiable or if information on sex or age was incomplete. Consequently, 2948 serum samples were included in the final dataset for the seroprevalence analysis.

### 2.2. Serological Testing

Serological testing for SFTSV antibodies was performed using a double-antigen sandwich ELISA, an established technique for detecting antibodies with 100% sensitivity and 99.57% specificity. This assay detects total SFTSV-specific antibodies, including immunoglobulin (Ig) G and IgM, indicating past or recent viral exposure.

In this study, a double-antigen ELISA was performed as an in-house assay in the laboratory following the methodology described previously [14]. Briefly, plates were coated with recombinant SFTSV nucleocapsid protein (rNP), followed by the addition of serum samples. Following incubation, HRP-conjugated rNP was introduced; a chromogenic reaction produced a measurable color change, indicating the presence of SFTSV-specific antibodies. Optical density was measured at 450 nm. A positive control and a negative control were each tested in quadruplicate per plate. During a two-month period, 36 plates were tested; the grand mean of the optical densities of the positive control was 1.253, and that of the negative control was 0.044. Precision performance characteristics were calculated as the within-run and within-laboratory percent coefficients of variation (%CV) [15]; within-run %CV represents intra-plate variability, and within-laboratory %CV reflects variabilities including intra- and inter-plate variability. Within-run %CVs for the positive and negative controls were 1.70% and 1.57%, respectively; within-laboratory %CVs were 5.40% and 3.73%, respectively. Results were expressed as a percentage of the positive control (PP), with the cutoff determined as the mean PP plus three standard deviations of the negative control [14]. Initially positive samples were all retested in duplicate and also positive in the retest.

### 2.3. Data Collection and Classification

Demographic data, including age, sex, and residential address, were retrieved from the biobank database. Geographical classification was based strictly on the patient’s documented residential address at the time of sample collection. This approach was taken to ensure that the regional seroprevalence reflects the patient’s actual place of residence and potential exposure, as patients in Korea occasionally visit tertiary hospitals located in different administrative regions.

Primary diagnoses were categorized into 10 analytic groups based on the International Classification of Diseases, 10th Revision (ICD-10) codes. Specific disease groups included gastric (C16), colorectal (C18–C20), hepatobiliary/pancreatic (C22–C25), lung (C34), breast (C50), prostate (C61), and thyroid cancers (C73), as well as diabetes (E11) and cardiovascular diseases (I10–I20). The ‘Others’ group consolidated health screening participants (n = 100), other less frequent cancers, and benign neoplasms. The definition of “chronic disease” was strictly based on the primary ICD-10 diagnosis code recorded at the time of biobank sample collection.

### 2.4. Statistical Analysis

Statistical analyses were performed using SAS version 9.4 (SAS Institute Inc., Cary, NC, USA) and R software version 4.4.1 (R Foundation for Statistical Computing, Vienna, Austria). The Chi-square test or Fisher’s exact test was used to compare seroprevalence between groups (sex, region, and disease type). The Cochran–Armitage test was used to analyze seropositivity trends across age groups. Statistical significance was set at *p* < 0.05. Seroprevalence and corresponding 95% confidence intervals (CIs) were estimated using the Clopper–Pearson exact method, which is appropriate for sparse data and extreme proportions.

To estimate independent associations while accounting for potential confounding, multivariable analyses were additionally conducted using Firth’s penalized logistic regression, which is appropriate for sparse data and low event counts and reduces small-sample bias. Variables examined in univariable analyses were included in the multivariable model based on epidemiological relevance, regardless of univariable statistical significance. Age was modeled as a continuous variable (per 10-year increase), and months were grouped into seasons to reduce model sparsity. Region was included as a fixed-effect covariate. Resulting adjusted odds ratios (aORs) and 95% CIs were calculated using the logistf package in R.

## 3. Results

### 3.1. Demographic Characteristics and Seroprevalence

Among the 2948 serum samples analyzed, 36 tested positive for SFTSV antibodies, yielding an overall seroprevalence of 1.22% (95% CI: 0.86–1.69%). Table 1 summarizes seroprevalence by sex and age group. The positivity rate was significantly higher in males (1.73%, 95% CI: 1.13–2.55%) than in females (0.73%, 95% CI: 0.36–1.30%) (*p* = 0.013). The mean age of the study population was 60.16 ± 14.24 years. Seroprevalence increased significantly with age (*p* = 0.001), peaking in the 70–79 age group (2.73%, 95% CI: 1.65–4.22%), followed by the ≥80 age group (1.53%) (Table 1).

### 3.2. Regional Seroprevalence

**Figure 1:**
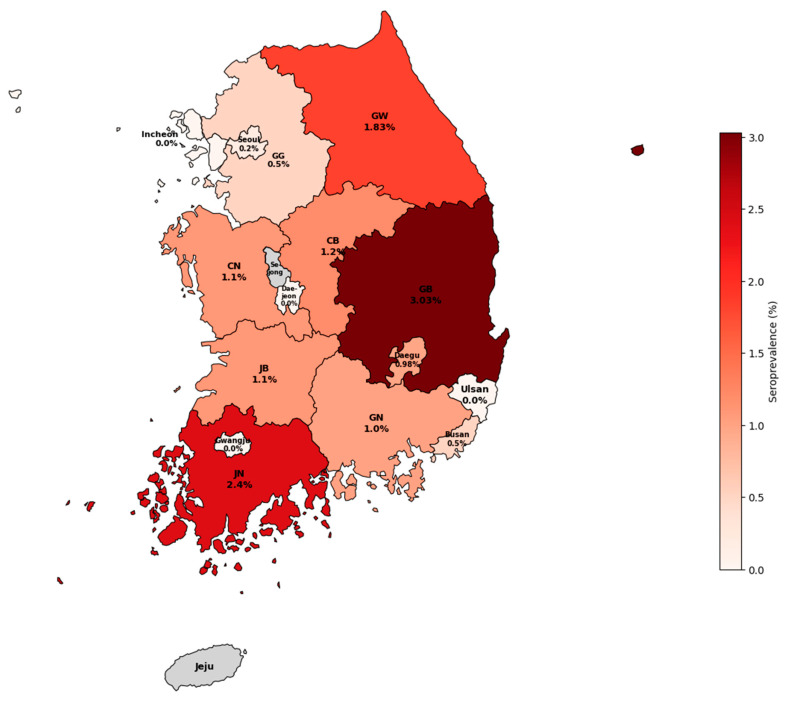
Geographical Distribution of SFTSV Seroprevalence. Rural regions, such as Gyeongbuk (GB) and Jeonnam (JN), showed higher seropositivity rates compared to urban areas, such as Seoul, Incheon, Ulsan, and Gwangju. The map highlights regional differences in seroprevalence across the mainland. Note: Jeju and Sejong are shown in gray as they were excluded from this analysis. Abbreviations: GB, Gyeongbuk; JN, Jeonnam; GW, Gangwon; GG, Gyeonggi; CB, Chungbuk; CN, Chungnam; JB, Jeonbuk; GN, Gyeongnam.

Geographical classification was based on the patient’s residential address. Regions known to be rural showed a higher seroprevalence. Gyeongbuk had the highest seropositivity (3.03%, 8/264), followed by Jeonnam (2.40%, 4/167) and Gangwon (1.83%, 13/709). Urban centers, such as Seoul, Busan, and Daegu, generally showed lower rates, although Daegu’s rate reached 0.98%. No positive cases were detected in Incheon, Gwangju, Daejeon, Ulsan, Gyeonggi, Chungnam, or Jeonbuk (Figure 1). No statistically significant differences were observed among regions (*p* = 0.181). Detailed analysis by sex within these regions are shown in Supplementary Table S1. Additionally, age-stratified analysis in high-seropositivity regions (Gangwon, Gyeongbuk, and Jeonnam) revealed that seropositivity was predominantly concentrated in the elderly population aged 70 years and older (Supplementary Table S2).

### 3.3. Seroprevalence by Underlying Disease (ICD Code)

Seroprevalence according to primary diagnosis is presented in Table 2. Among the cancer groups, patients with hepatobiliary/pancreatic cancer showed the highest seropositivity rate (3.16%, 95% CI: 0.66–8.95%), followed by those with prostate cancer (2.50%, 95% CI: 0.30–8.74%) and lung cancer (1.61%, 95% CI: 0.59–3.47%). In the hepatobiliary/pancreatic group, positive cases were predominantly observed in the 70–79 age group (8.11% within that age bracket). For prostate cancer, positive cases were found in older adults (60 s and 70 s). There were no statistically significant differences in seroprevalence among the disease categories (Table 2).

### 3.4. Seasonal Distribution of Seropositive Samples

Analysis of sample collection dates revealed statistically significant seasonal variation in seropositivity (Table 3; *p* = 0.002). The highest number of positive samples occurred in specimens collected during autumn, specifically October (3.23%, 7/217) and September (3.02%, 7/232), followed by July (2.20%, 6/273) and April (2.04%, 4/196). Conversely, no positive cases were detected in the samples collected in January, March, and May.

### 3.5. Multivariable Analysis of Factors Associated with Seropositivity

In multivariable Firth’s penalized logistic regression analysis, increasing age and autumn season were independently associated with higher odds of seropositivity, after adjustment for residential area and other covariates. Although region was not independently associated with seropositivity after adjustment, it was retained in the model to account for geographic differences in exposure risk.

Results showed older age (aOR 1.47 per 10-year increase, 95% CI 1.12–1.97) as the strongest independent predictor of seropositivity (Table 4). Notably, winter sampling showed substantially lower odds compared to autumn (aOR 0.10, 95% CI 0.02–0.33), followed by spring (aOR 0.25, 95% CI 0.08–0.65).

## 4. Discussion

This study provides a large-scale retrospective analysis of SFTS seroprevalence among patients with chronic diseases using South Korean biobank samples. In this nationwide cohort, we identified an overall seroprevalence of 1.22%. This finding can be contextualized by comparison with previous seroepidemiological studies in South Korea that primarily focused on high-risk groups or specific regions. For instance, Han et al. reported a 4.1% seroprevalence in rural areas [10] and Yoo et al. identified a rate of 2.4% among the agricultural population of Jeju Island [11]. Similarly, a study conducted in southeastern Korea, a region known for its high SFTS incidence, reported a seropositivity of approximately 2.1% in patients visiting a local tertiary hospital [16]. At the national level, a recent study using Korea National Health & Nutrition Examination Survey (KNHANES) specimens reported a 2.1–4.1% seropositivity in rural populations [17]. Although our observed rate (1.22%) was lower than those of these high-risk or rural-specific cohorts, it was comparable with the overall national rates and higher than those typically found in healthy urban donors. Consistent with our findings, a recent study on Jeju Island reported a seroprevalence of 1.7% in the general population, indicating persistent exposure to endemic regions [12]. In China, a systematic review and meta-analysis reported a pooled seroprevalence of 4.3% in a healthy population [18], noticeably higher than the 1.22% observed in our chronic disease cohort, likely attributable to the higher endemicity in rural China or demographic differences, as our study specifically targeted patients with underlying conditions rather than the general rural population. These comparisons suggest that, while the risk is highest in agricultural communities, it extends significantly to the chronic disease population investigated in this study.

Our findings confirm well-established epidemiological risk factors for SFTS. In univariable analysis, males showed significantly higher seroprevalence than females. However, multivariable analysis revealed that this sex difference was not statistically significant after adjusting for age and region. This suggests that the higher seroprevalence observed in males may be largely driven by demographic factors, such as a higher proportion of elderly males residing in rural, high-risk areas, or by behavioral patterns associated with rural living, rather than biological susceptibility [4,5,10,17]. Older age remained a robust risk factor. Rural regions, such as Gangwon, Gyeongbuk, and Jeonnam, exhibited higher seroprevalence, consistent with previous reports identifying these areas as high-risk zones [3,6,17].

A novel finding of this study is the seroprevalence breakdown by underlying disease. Although the differences were not statistically significant due to the limited number of positive cases, we observed relatively higher seropositivity in patients with hepatobiliary/pancreatic (3.16%) and prostate (2.50%) cancers. The observation regarding prostate cancer likely reflects the demographic profile of this malignancy, which predominantly affects older males—a group with established high risk for outdoor tick exposure—rather than a direct association with the cancer itself. Similarly, high rates in patients with hepatobiliary cancer may be attributable to shared environmental risk factors among rural residents, who may represent a larger proportion of the patients visiting the regional hospitals participating in this study. Furthermore, the presence of antibodies against SFTSV in immunocompromised or chronically ill patients is clinically relevant. Although total antibodies indicate past exposure, these individuals represent a vulnerable group where new or repeat infections could be exacerbated by their underlying conditions [13].

Notably, the samples collected in September and October had the highest positivity rates. However, the date of sample collection does not necessarily reflect the time of exposure, especially given that IgG antibodies can persist for years [19,20]. Given the lack of clinical data on acute symptoms or IgM status in biobank samples, the observed seasonal variation warrants cautious interpretation and may reflect healthcare-seeking behaviors rather than actual transmission dynamics.

This study has several limitations. First, as samples were obtained from hospital-based biobanks, the findings apply specifically to “biobank-based chronic disease patients” and may not fully represent the general population due to potential selection bias. Second, detailed occupational and outdoor activity histories were unavailable. Third, despite performing multivariable analysis, the relatively small number of positive cases warrants caution in interpretation. Fourth, our assay detects total antibodies (including IgG and IgM). Since SFTSV-specific IgG antibodies can persist for years while IgM declines rapidly [19,20], this method cannot distinguish between recent and past infections. Consequently, the observed seasonal peaks may not necessarily reflect acute transmission dynamics. Fifth, positive results were not directly confirmed by the neutralization test due to the unavailability of a BSL-3 facility. However, the ELISA used in this study has been previously validated with high sensitivity (100%) and specificity (99.57%) against neutralization tests [14]. Further prospective research is warranted to clarify the prognostic implications of SFTS in patients with chronic underlying conditions. Integrating clinical parameters, such as severity, will provide a more comprehensive understanding of the disease burden. These insights are vital for developing targeted preventive measures and education for high-risk groups.

## 5. Conclusions

We identified a 1.22% SFTSV antibody seroprevalence in a nationwide cohort of patients with chronic diseases. Older age was identified as the primary independent risk factor. While seroprevalence was notably higher in residents of rural provinces and specific cancer groups (e.g., hepatobiliary and prostate cancer), these findings likely reflect overlapping demographic characteristics with high-risk populations rather than independent disease-specific susceptibility. These findings indicate that patients with chronic diseases, particularly the elderly in rural areas, may have a non-negligible history of SFTSV infection. Hence, public health education regarding tick avoidance should be actively integrated into routine clinical care for these vulnerable patient groups.

## Figures and Tables

**Table 1:** Seroprevalence of SFTSV Antibodies by Sex and Age Group.

Characteristic	Total (n)	Positive (n)	Seropositivity, % (95% CI)	*p*-Value
Sex				0.013 ^a^
Male	1441	25	1.73 (1.13–2.55)	
Female	1507	11	0.73 (0.36–1.30)	
Age Group (years)				0.001 ^b^
<30	73	1	1.37 (0.03–7.40)	
30–39	194	1	0.52 (0.01–2.84)	
40–49	408	1	0.25 (0.01–1.36)	
50–59	673	5	0.74 (0.24–1.73)	
60–69	707	6	0.85 (0.31–1.84)	
70–79	697	19	2.73 (1.65–4.22)	
≥80	196	3	1.53 (0.32–4.41)	
Total	2948	36	1.22 (0.86–1.69)	

Note: ^a^ Chi-square test; ^b^ Cochran–Armitage test for trend.

**Table 2:** Seroprevalence of SFTSV Antibodies by Disease Category.

Disease Category	Total (n)	Male/Female (Ratio)	Mean Age ± SD	Positive (n)	Seropositivity, % (95% CI)
Hepatobiliary/Pancreatic Cancer	95	2.96	65.4 ± 11.8	3	3.16 (0.66–8.95)
Prostate Cancer	80	-	70.6 ± 5.3	2	2.50 (0.30–8.74)
Lung Cancer	373	2.89	68.5 ± 9.1	6	1.61 (0.59–3.47)
Gastric Cancer	512	2.05	64.5 ± 11.7	8	1.56 (0.68–3.06)
Breast Cancer	216	-	54.7 ± 12.1	3	1.39 (0.29–4.01)
Colorectal Cancer	376	1.47	66.8 ± 11.6	5	1.33 (0.43–3.08)
Diabetes	300	1.05	60.4 ± 12.3	4	1.33 (0.36–3.38)
Thyroid Cancer	565	0.14	47.2 ± 11.7	4	0.71 (0.19–1.80)
Cardiovascular Diseases	311	1.21	64.3 ± 11.7	1	0.32 (0.01–1.78)
Others (Benign/Screening)	120	0.69	43.1 ± 15.4	0	0.00 (0.00–3.03)

**Table 3:** Monthly Seroprevalence of SFTSV Antibodies.

Month	Total (n)	Positive (n)	Seropositivity, % (95% CI)
January	325	0	0.00 (0.00–1.13)
February	244	1	0.41 (0.01–2.26)
March	209	0	0.00 (0.00–1.75)
April	196	4	2.04 (0.56–5.14)
May	187	0	0.00 (0.00–1.95)
June	242	2	0.83 (0.10–2.95)
July	273	6	2.20 (0.81–4.72)
August	330	3	0.91 (0.19–2.63)
September	232	7	3.02 (1.22–6.12)
October	217	7	3.23 (1.31–6.53)
November	251	5	1.99 (0.65–4.59)
December	242	1	0.41 (0.01–2.28)
Total	2948	36	1.22 (0.86–1.69)

Note: *p* = 0.002.

**Table 4:** Multivariable Logistic Regression Analysis of Risk Factors for SFTSV Seropositivity.

Characteristic	Adjusted OR, 95% CI	*p*-Value
Sex		
Male	(Ref.)	
Female	0.52 (0.25–1.05)	0.068
Age Group (per 10-year increase)	1.47 (1.12–1.97)	0.005
Season		
Spring	0.25 (0.08–0.65)	0.003
Summer	0.50 (0.23–1.03)	0.062
Autumn	(Ref.)	
Winter	0.10 (0.02–0.33)	<0.001
Urban/Rural		
Urban	(Ref.)	
Rural	0.77 (0.33–2.16)	0.600

Note: OR = Odds Ratio, CI = Confidence Interval, (Ref.) = Reference group.

## Data Availability

The data presented in this study are available on request from the corresponding author. The data are not publicly available due to ethical restrictions imposed by the Institutional Review Board of Pusan National University Hospital (Approval No. 1810-029-072), which prohibit unrestricted public sharing to protect participant confidentiality.

## Supplementary Materials

The following supporting information can be downloaded at: https://www.mdpi.com/article/10.3390/v18020217/s1, Table S1: Detailed SFTSV seroprevalence by region and sex; Table S2: Seroprevalence by age group in regions with high seroprevalence.

## Abbreviations

The following abbreviations are used in this manuscript:

ELISA	Enzyme-linked immunosorbent assay
ICD	International Classification of Diseases
Ig	Immunoglobulin
KBN	Korea Biobank Network
PP	Percentage of the positive control
rNP	Recombinant nucleocapsid protein
SFTS	Severe fever with thrombocytopenia syndrome
SFTSV	Severe fever with thrombocytopenia syndrome virus
