# Supplementary material for: Seroprevalence and Epidemiological Characteristics of Severe Fever with Thrombocytopenia Syndrome in Patients with Chronic Diseases in Korea
Source: Viruses. 2026 Feb 6;18(2):217. doi: 10.3390/v18020217 (PMC12944934; doi:10.3390/v18020217)
Supplement: Supplementary file 1 [file viruses-18-00217-s001.zip › viruses-4088045-supplementary.pdf]

Supplementary Table S1. Detailed SFTSV Seroprevalence by Region and Sex

Region	Category	Mean Age ± SD	Total (N)	Positive (N)	Seropositivity, % (95% CI)
**Seoul**	Total	62.8 ± 12.0	101	1	0.99 (0.03-5.39)
	Male	64.3 ± 11.2	67	1	1.49 (0.04-8.04)
	Female	59.7 ± 13.0	34	0	0.00 (0.00-10.28)
**Busan**	Total	56.5 ± 9.2	2	0	0.00 (0.00-84.19)
	Male	50.0	1	0	0.00 (0.00-97.50)
	Female	63.0	1	0	0.00 (0.00-97.50)
**Daegu**	Total	54.1 ± 13.9	612	6	0.98 (0.36-2.12)
	Male	58.1 ± 13.8	206	5	2.43 (0.79-5.57)
	Female	52.1 ± 13.5	406	1	0.25 (0.01-1.36)
**Incheon**	Total	62.0 ± 17.6	9	0	0.00 (0.00-33.63)
	Male	65.2 ± 10.3	6	0	0.00 (0.00-0.93)
	Female	55.7 ± 29.7	3	0	0.00 (0.00-70.76)
**Gwangju**	Total	57.9 ± 14.6	134	0	0.00 (0.00-2.72)
	Male	63.9 ± 13.1	69	0	0.00 (0.00-5.21)
	Female	51.6 ± 13.5	65	0	0.00 (0.00-5.52)
**Daejeon**	Total	44.5 ± 9.7	75	0	0.00 (0.00-4.80)
	Male	50.5 ± 2.1	2	0	0.00 (0.00-84.19)
	Female	44.3 ± 9.8	73	0	0.00 (0.00-4.93)
**Ulsan**	Total	53.0	1	0	0.00 (0.00-97.50)
	Male	-	0	0	N/A
	Female	53.0	1	0	0.00 (0.00-97.50)
**Sejong**	Total	62.4 ± 14.6	11	0	0.00 (0.00-28.49)
	Male	62.1 ± 14.4	7	0	0.00 (0.00-40.96)
	Female	62.8 ± 17.1	4	0	0.00 (0.00-60.24)
**Gyeonggi**	Total	55.2 ± 16.7	117	0	0.00 (0.00-3.10)
	Male	59.7 ± 13.4	64	0	0.00 (0.00-5.60)
	Female	49.8 ± 18.6	53	0	0.00 (0.00-6.72)
**Gangwon**	Total	67.1 ± 12.2	709	13	1.83 (0.98-3.12)
	Male	68.1 ± 10.5	429	10	2.33 (1.12-4.24)
	Female	65.4 ± 14.3	280	3	1.07 (0.22-3.10)
**Chungbuk**	Total	63.4 ± 11.8	369	1	0.27 (0.01-1.50)
	Male	61.7 ± 12.1	204	0	0.00 (0.00-1.79)
	Female	65.4 ± 11.2	165	1	0.61 (0.02-3.33)
**Chungnam**	Total	54.9 ± 14.3	31	0	0.00 (0.00-11.22)
	Male	61.0 ± 13.8	11	0	0.00 (0.00-28.49)
	Female	51.6 ± 13.8	20	0	0.00 (0.00-16.84)
**Jeonbuk**	Total	57.5 ± 14.5	57	0	0.00 (0.00-6.27)
	Male	64.5 ± 10.1	23	0	0.00 (0.00-14.82)
	Female	52.8 ± 15.2	34	0	0.00 (0.00-10.28)
**Jeonnam**	Total	63.5 ± 12.6	167	4	2.40 (0.66-6.02)
	Male	65.8 ± 10.6	105	3	2.86 (0.59-8.12)
	Female	59.7 ± 14.8	62	1	1.61 (0.04-8.66)
**Gyeongbuk**	Total	55.3 ± 14.1	264	8	3.03 (1.32-5.88)
	Male	60.0 ± 12.9	103	5	4.85 (1.59-10.97)
	Female	52.3 ± 14.1	161	3	1.86 (0.39-5.35)
**Gyeongnam**	Total	61.6 ± 12.6	289	3	1.04 (0.21-3.00)
	Male	63.0 ± 12.6	144	1	0.69 (0.02-3.81)
	Female	60.2 ± 12.6	145	2	1.38 (0.17-4.89)

Supplementary Table S2. Seroprevalence by Age Group in Regions with high seroprevalence

Region	Age Group	Total (N)	Positive (N)	Seropositivity, % (95% CI)
**Gangwon**	<30	3	0	0.00 (0.00‑70.76)
	30–39	10	1	10.00 (0.25‑44.50)
	40–49	55	0	0.00 (0.00‑6.49)
	50–59	124	1	0.81 (0.02‑4.41)
	60–69	163	1	0.61 (0.02‑3.37)
	70–79	253	9	3.56 (1.64‑6.65)
	≥80	101	1	0.99 (0.03‑5.39)
**Gyeongbuk**	<30	11	0	0.00 (0.00‑28.49)
	30–39	27	0	0.00 (0.00‑12.77)
	40–49	48	0	0.00 (0.00‑7.40)
	50–59	75	3	4.00 (0.83‑11.25)
	60–69	58	2	3.45 (0.42‑11.91)
	70–79	36	2	5.56 (0.68‑18.66)
	≥80	9	1	11.11 (0.28‑48.25)
**Jeonnam**	<30	0	0	N/A
	30–39	9	0	0.00 (0.00‑33.63)
	40–49	15	0	0.00 (0.00‑21.80)
	50–59	34	1	2.94 (0.07‑15.33)
	60–69	38	1	2.63 (0.07‑13.81)
	70–79	62	2	3.23 (0.39‑11.17)
	≥80	9	0	0.00 (0.00‑33.63)
